# Poster Session II - A291 THE ENVIRONMENTAL IMPACT OF VIRTUAL SPECIALIST CARE AMONG INDIVIDUALS WITH INFLAMMATORY BOWEL DISEASE IN ONTARIO

**DOI:** 10.1093/jcag/gwaf042.290

**Published:** 2026-02-13

**Authors:** L J Nguyen, V W Huang, C Chong, D McCormack, P Habashi, P Tandon

**Affiliations:** Health Sciences, Queen’s University, Kingston, ON, Canada; University of Toronto, Toronto, ON, Canada; Health Sciences, Queen’s University, Kingston, ON, Canada; ICES, Toronto, ON, Canada; Lunenfeld-Tanenbaum Research Institute, Toronto, ON, Canada; University Health Network, Toronto, ON, Canada

## Abstract

**Background:**

Transportation to and from healthcare appointments contributes substantially to healthcare-related greenhouse gas emissions. During the COVID-19 pandemic, widespread adoption of virtual care transformed healthcare delivery. However, the environmental benefits of this transition remain poorly quantified in the Canadian context, particularly for chronic conditions such as inflammatory bowel disease (IBD) that require ongoing specialist follow-up.

**Aims:**

Our aim was to estimate the travel distance and associated reduction in transportation-related carbon emissions attributable to virtual IBD specialist visits in Ontario during the first year of the COVID-19 pandemic.

**Methods:**

We used administrative databases at ICES, Ontario. Individuals in the Ontario Crohn’s and Colitis Cohort between April 1, 2020 and March 31, 2021 were included. Specialist visits were classified as in-person or virtual using Ontario Health Insurance Plan fee codes. Each patient’s residential postal code was linked to their IBD specialist’s office location through the ICES Physician Database. The round-trip distance between the patient’s residence and the clinic was calculated for every virtual visit to represent kilometers of travel avoided. Total avoided distance was converted into carbon dioxide (CO_2_) emissions using an emission factor of 206 g CO_2_ per kilometer, reflecting average passenger-vehicle emissions in Canada.

**Results:**

A total of 98,099 adults with IBD in Ontario were identified. The cohort was 48% male and 52% female, and 11% lived in rural regions of Ontario. Ninety-two percent of all IBD specialist visits were virtual during the study period. The number of kilometers of travel avoided, and reductions in carbon emissions are shown in Table 1. The cumulative travel distance avoided through virtual visits was estimated at 6.7 million kilometers, corresponding to approximately 1.38 million kilograms of CO_2_ emissions prevented.

**Conclusions:**

The rapid uptake of virtual IBD specialist care during the COVID-19 pandemic led to a measurable reduction in transportation-related carbon emissions across Ontario. These findings highlight the potential for virtual healthcare to advance environmental sustainability goals while maintaining continuity of care for patients with chronic disease. As healthcare systems consider post-pandemic models of care, integrating environmental metrics into virtual care policy planning may support Canada’s broader climate action objectives.

**Funding Agencies:**

CCCThe Leona M. and Harry B. Hemlsley Charitable Trust

**Table:** A291 Table 1:

	Quarter 1	Quarter 2	Quarter 3	Quarter 4
Mean travel distance avoided (km, SD)	68.1 (141)	68.3 (147)	67.2 (142)	70.9 (158)
Median travel distance avoided (km, IQR)	25.4 (55)	25.4 (55)	25.5 (56)	26.7 (59)
Mean reduction in CO2 (kg, SD)	14.0 (29.0)	14.0 (30.3)	13.8 (29.2)	14.6 (32.5)
Median reduction in CO2 (kg, IQR)	5.2 (11.4)	5.2 (11.3)	5.2 (29.5)	5.5 (12.1)
Total reduction in CO2 (million kg)	367	324	333	358
Cumulative total reduction in CO2 (million kg)	367	691	1,025	1,383

